# Landscape of the Peripheral Immune Response Induced by Local Microwave Ablation in Patients with Breast Cancer

**DOI:** 10.1002/advs.202200033

**Published:** 2022-04-11

**Authors:** Wenbin Zhou, Muxin Yu, Xinrui Mao, Hong Pan, Xinyu Tang, Ji Wang, Nan Che, Hui Xie, Lijun Ling, Yi Zhao, Xiaoan Liu, Cong Wang, Kai Zhang, Wen Qiu, Qiang Ding, Shui Wang

**Affiliations:** ^1^ Department of Breast Surgery The First Affiliated Hospital with Nanjing Medical University 300 Guangzhou Road Nanjing 210029 China; ^2^ Jiangsu Key Lab of Cancer Biomarkers, Prevention and Treatment, Jiangsu Collaborative Innovation Center for Cancer Personalized Medicine, School of Public Health Nanjing Medical University Nanjing 211166 China; ^3^ Department of Rheumatology and Immunology The First Affiliated Hospital with Nanjing Medical University 300 Guangzhou Road Nanjing 210029 China; ^4^ Department of Pathology The First Affiliated Hospital with Nanjing Medical University 300 Guangzhou Road Nanjing 210029 China; ^5^ Pancreas Center & Department of General Surgery The First Affiliated Hospital with Nanjing Medical University Nanjing Jiangsu 210029 China; ^6^ Pancreas Institute of Nanjing Medical University Nanjing Jiangsu 210029 China; ^7^ Department of Immunology and Key Laboratory of Immunological Environment and Disease Nanjing Medical University Nanjing 211166 China; ^8^ Key Laboratory of Antibody Technology of Ministry of Health Nanjing Medical University Nanjing Jiangsu 211166 China

**Keywords:** breast cancer, immune response, microwave ablation, T cells

## Abstract

Minimally invasive thermal therapies have been attempted in the treatment of breast cancer, and the immune response induced by these therapies has not been fully reported. A clinical trial is performed to determine the effect of microwave ablation (MWA) in the treatment of early‐stage breast cancer. The authors perform single‐cell RNA sequencing on peripheral blood mononuclear cells (PBMCs) from six patients before and after ablation. NK and CD8^+^T cells are activated by MWA of breast cancer, with the increased inhibitory signature of CD8^+^T cells but not dysfunctional. Enhanced co‐stimulatory signature of CD4^+^ T cells is observed and increased frequency of ICOS^+^CD4^+^ T cells after MWA is confirmed by flow cytometric analysis. After ablation, T‐cell clones expand with increased T‐cell receptor diversities. Activated antigen receptor‐mediated signaling pathways are found in B cells. Enhanced interactions between B cells and CD4^+^ T cells are found, indicating that B cells are important antigen‐presenting cells that initiate CD4^+^T cells in MWA‐induced immune response. Blockade of CTLA‐4 or PD‐1 of post‐MWA PBMCs show higher T‐cell activity than that of pre‐MWA PBMCs. This study provide global characteristics of MWA‐induced systemic immune response and pave a way for the identification of potential targets to improve the immune response.

## Graphical abstract

This study finds that peripheral NK and T cells are activated by MWA of breast cancer partly dependent on B cell and CD4^+^ T cell collaboration, and MWA combined with immune checkpoint inhibitor may be a promising strategy for the treatment of early‐stage breast cancer.

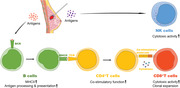

## 1 Introduction

More patients with early‐stage breast cancer have been diagnosed because of the development of screening techniques.^[^ ^1^ ^]^ The surgical treatment of early breast cancer has evolved from mastectomy to breast‐conserving surgery, and there is a growing trend to apply minimally invasive thermal therapy to ablate the tumor with the ultimate goal of omitting the surgical excision of the primary tumor.^[^ ^2^ ^]^ Importantly, the survival of these patients has been obviously improved, since the advances in systemic therapies. However, several patients still develop metastatic disease, especially for triple‐negative breast cancer (TNBC), and there is still room to improve the survival.^[^ ^3^ ^]^ Recently, immunotherapy in combination with chemotherapy has demonstrated efficacy for the treatment of advanced/metastatic TNBC, and ongoing clinical trials are investigating immunotherapy in early‐stage breast cancer.^[^ ^4^ ^]^ As an immunologically “cold” tumor, combinatorial strategy is urgently needed to improve the effect of immunotherapy.^[^ ^5^ ^]^

Minimally invasive therapies, including radiofrequency ablation (RFA), microwave ablation (MWA), cryotherapy, high‐intensity focused ultrasound, and laser therapy, have been attempted in the treatment of early‐stage breast cancer.^[^ ^2^, ^6^ ^]^ The feasibility studies^[^ ^7^ ^]^ have showed high complete ablation rates, and favorable local control has been reported in limited long‐term studies.^[^ ^8^ ^]^ Besides, immune response after thermal ablation of solid tumor has been reported in preclinical and clinical studies.^[^ ^6^, ^9^ ^]^ In situ tumor ablation can create an antigen source,^[^ ^10^ ^]^ and these tumor specific antigens are presented to lymphocytes by dendritic cells (DCs) and macrophages.^[^ ^11^ ^]^ Weak adaptive immune response has been mostly reported after RFA in the treatment of solid tumors.^[^ ^12^ ^]^ Moreover, NK cell response has been reported in limited studies.^[^ ^11^, ^13^ ^]^ As an effective local therapy, minimally invasive thermal therapy may be a trigger of antitumor immunity, and its combination with immunotherapy may be a promising strategy in the treatment of early‐stage breast cancer.^[^ ^14^ ^]^

Due to the advantages in comparison to other techniques,^[^ ^15^ ^]^ MWA has been reported in the treatment of early‐stage breast cancer, with a high complete ablation rate.^[^ ^7^, ^14^, ^16^ ^]^ Interestingly, MWA in the treatment of breast cancer induces Th1‐type immune response and elevated proportions of activated NK cells in limited cases, unlike the systemic inflammatory and immunosuppressive microenvironments induced by surgery.^[^ ^11^, ^14^, ^17^ ^]^ To the best of our knowledge, the landscape of thermal ablation‐induced immune response has not been reported. Single‐cell RNA sequencing (scRNA‐seq) allows for comprehensive profiling of the immune system in an unprecedented way, providing potential target for immunotherapy as a new strategy.^[^ ^18^ ^]^ To find key cellular subsets and underlying mechanism of MWA‐induced immune response in the treatment of early‐stage breast cancer, we applied scRNA‐seq to comprehensively characterize the immune response in peripheral blood mononuclear cells (PBMCs) from 6 patients before and after MWA. It revealed that, (1) global characteristics of systemic anti‐tumor immune response induced by MWA in the treatment of breast cancer was firstly reported; (2) B cells were important antigen‐presenting cells (APCs) that initiate CD4^+^T cells in MWA‐induced immune response; (3) based on the comprehensive characteristics of MWA‐induced response, we found that immune checkpoint inhibitors synergistically activated peripheral T cells after MWA in vitro.

## 2 Results

### 2.1 Study Design and Analysis of Single Immune Cell Profiling

**Figure 1:**
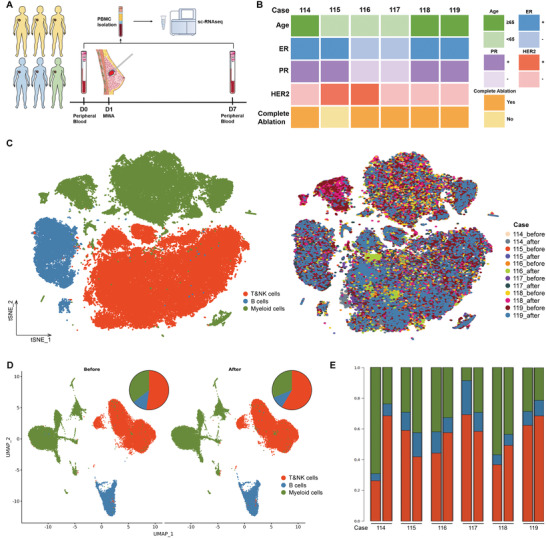
ScRNA‐seq profiling of peripheral immune response induced by MWA of early‐stage breast cancer. A) Schematic representation of the scRNA‐seq strategy. B) Basic characteristics of enrolled patients. C) T‐distributed stochastic neighbor embedding (t‐SNE) plot, showing cell types and cell origins by patients (*n* = 6). D) Uniform manifold approximation and projection (UMAP) plot, showing dynamic changes of peripheral immune cells induced by MWA of breast cancer (*n* = 6). E) Histogram indicating the proportion of peripheral immune cells of each patient before (the first) and after (the second) MWA.

A clinical trial (ChiCTR2000029155) was performed to determine the local effect of MWA in the treatment of early‐stage breast cancer. To resolve the landscape of the systemic immune response induced by local MWA in patients with early‐stage breast cancer, we collected the peripheral blood before and one week after MWA from 6 patients with early‐stage breast cancer and performed scRNA‐seq (**Figure** **1**A). Of these 6 patients (Figure 1B), 3 had ER^+^/HER2‐ subtype, 2 had HER2^+^, and 1 had TNBC. Because of obvious pain during the procedure, the prescheduled MWA was ended earlier in one case, and complete ablation was not observed in this case (Figure 1B). After standard data processing and quality control procedures, we obtained transcriptomic profiles for 82,473 cells.

### 2.2 An Overview of Myeloid, B, T, and NK Cells in the Blood

Using t‐distributed stochastic neighbor embedding (t‐SNE), three major distinct cell clusters, including myeloid, NK and T, and B cells, were identified (Figure 1C). When 2227 undefined cells were excluded, 80,246 scRNA‐seq profiles were classified, including 26,675 myeloid cells, 44,442 NK and T cells, and 9129 B cells. Then, the cells of each lineage were clustered separately, and a total of 25 immune cell clusters were identified across all patients before and after MWA.

After MWA, patients had increased proportion of NK and T cells, and decreased proportion of myeloid cells, with stable levels of B cells in comparison to that before MWA in peripheral blood (Figure 1D), with similar changing regularity when any one case was left out (Table S1, Supporting Information). Importantly, most patients showed the same changing regularity induced by MWA (Figure 1E), including three luminal cases and one HER2 positive case, with enhanced proportion of NK and T cells.

### 2.3 Activated Peripheral NK Cells Induced by MWA of Breast Cancer

**Figure 2:**
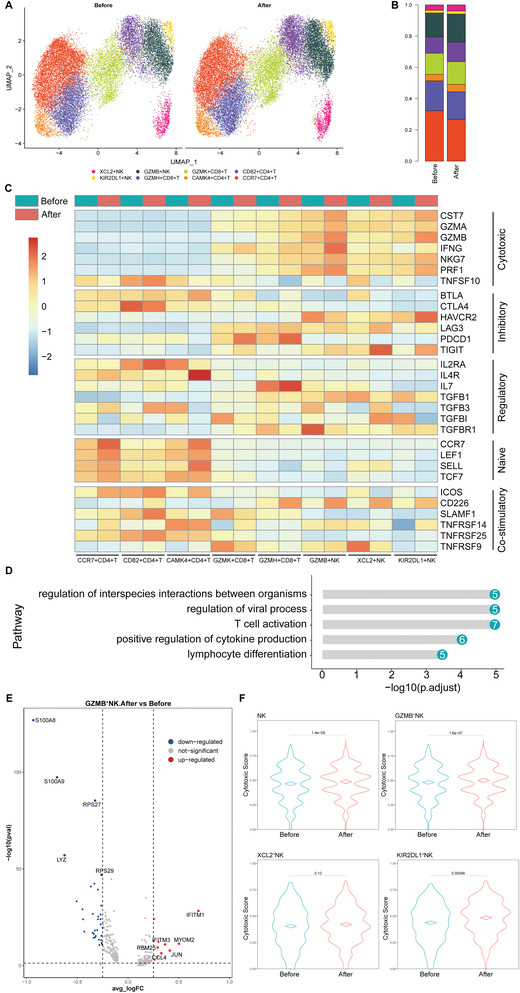
Overview changes of the NK and T cells, and the activated phenotypes of NK cells induced by MWA of breast cancer (*n* = 6). A) UMAP analysis of peripheral NK and T cells showing 8 clusters before and after MWA. B) The proportions of each cell cluster before and after ablation. C) Heatmap of the gene sets of cytotoxicity, exhaustion/inhibitory, regulatory, naïve and co‐stimulation of these 8 cell clusters. D) GO enrichment pathway analysis of genes preferentially upregulated in XCL2^+^NK cells. E) Volcano plot showing upregulated genes of peripheral GZMB^+^NK cells induced by MWA. F) The cytotoxic scores of different peripheral NK cell clusters before and after MWA.

Innate immunity serves as the first line of defense against cancer. NK cells are effector lymphocytes of the innate immune system, providing transient protection against cancer; Moreover, NK cells can boost the maturation and activation of T cells, through a combination of cell surface receptors and cytokines. To further understand the NK and T cell compartments of all enrolled patients, we re‐clustered those cells and identified 8 distinct cell types, including 3 NK cell clusters and 5 T cell clusters (**Figure** **2**A).

For NK cells (Figure S1, Supporting Information), 3 clusters were identified, including GZMB^+^NK cell (NK_c1), KIR2DL1^+^NK cell (NK_c2), and XCL2^+^NK cell (NK_c3). Cells of the first NK cell cluster, NK_c1, were characterized by the high expression of GZMB, GNLY, PRF1, KLRD1, SPON2, and FCER1G, indicative of high cytotoxic activities. The second cluster, NK_c2, specifically expressed killer cell immunoglobulin‐like receptors (KIRs) including KIR2DL1, KIR2DS4, and KIR3DL1. The third cluster, NK_c3, specifically expressed XCL2 and XCL1, indicative of high chemokines function. After MWA, patients had increased proportions of GZMB^+^NK cell (NK_c1), although no significant difference was observed (Figure 2B). Of these cell clusters, the selected gene expression of different functions was shown (Figure 2C).

Compared to that before MWA, gene ontology (GO) enrichment analysis indicated XCL2^+^NK cells after MWA were specifically enriched in genes associated with cytokine production pathway, lymphocyte differentiation pathway, and T cell activation pathway (Figure 2D), indicating higher chemokines function of XCL2^+^ NK cells induced by MWA.

Compared with the expression level before MWA (Figure 2E), upregulation of IFITM1, MYOM2, JUN, IFITM3, CCL4, and RBM25 was observed in GZMB^+^NK cells after MWA, indicating higher cytotoxic activity induced by MWA in this cytotoxic NK cell cluster.

To further determine the cytotoxic function of NK cells, the cytotoxic score^[^ ^19^ ^]^ was evaluated before and after ablation. We found that the cytotoxic function of NK cells after MWA was significantly higher than that before ablation (Figure 2F), especially for GZMB^+^NK cells (NK_c1) and KIR2DL1^+^NK cells (NK_c2).

### 2.4 Peripheral CD8^+^T Cells Were Activated by MWA of Breast Cancer

The interplay of innate and adaptive immunity is complex. In contrast to innate immunity, adaptive immunity can provide long‐term protection against cancer after activation. Above results suggested that NK cells were activated by MWA of breast cancer. After complete ablation of the primary tumor, T cell response may prevent recurrence of early‐stage breast cancer. To determine the MWA‐induced T cell response, 5 clusters were identified, including CCR7^+^CD4^+^T cell (CD4_c1), CD82^+^CD4^+^T cell (CD4_c2), CAMK4^+^CD4^+^T cell (CD4_c3), GZMK^+^CD8^+^T cell (CD8_c1), and GZMH^+^CD8^+^T cell (CD8_c2). The first CD8^+^ T cell cluster, GZMK^+^CD8^+^T cell (CD8_c1), was characterized by the high expression of GZMK, KLRG1, CD69, TRGC2, CXCR4, and CCL5, and considered as effector memory CD8^+^ T cells. The second cluster, GZMH^+^CD8^+^T cell (CD8_c2), with high expression of TRGC2, GZMH, FGFBP2, NKG7, CCL5, GNLY, KLRG1, GZMA, and GZMB, was recognized as cytotoxic CD8^+^ T cells.

CD8^+^T lymphocytes are the most potent killers in the cell‐mediated anti‐tumor immune response arsenal. To determine the MWA‐induced T cell response, we compared peripheral CD8^+^ T cell responses before and after MWA. After MWA, patients had increased proportions of GZMK^+^CD8^+^T cell (CD8_c1), and GZMH^+^CD8^+^T cell (CD8_c2), although no significant difference was observed (Figure 2B).

**Figure 3:**
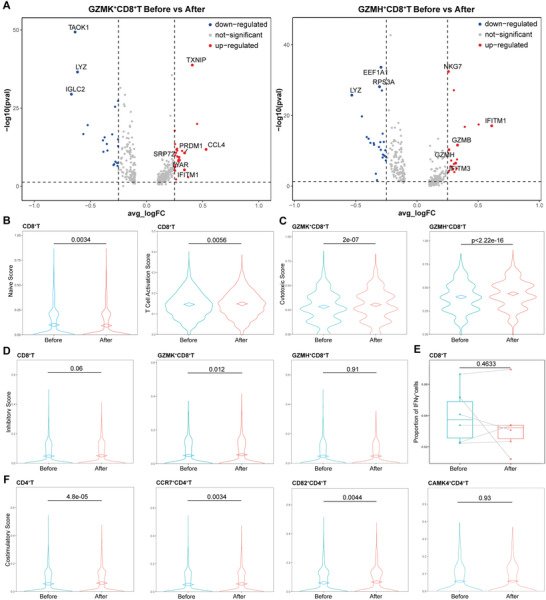
The activated peripheral T cells induced by MWA of breast cancer (*n* = 6). A) Volcano plot showing upregulated genes of peripheral GZMK^+^CD8^+^T cells and GZMH^+^CD8^+^T cells induced by MWA of breast cancer. The biological scores before and after MWA, including B) naïve, B) activation, C) cytotoxic, D) inhibitory, and F) costimulatory scores in specific cell clusters. E) The proportion of IFN‐*γ* ^+^CD8^+^T cells in peripheral CD8^+^ T cells before and after ablation (*n* = 6).

For GZMK^+^CD8^+^T cell cluster, MWA induced upregulation of CCL4, TXNIP, PRDM1, IFITM1, LYAR, and SRP72, indicating increased chemokine activity induced by MWA (**Figure** **3**A). Compared with the expression level before MWA (Figure 3A), upregulation of cytotoxic genes, including IFITM1, TRGC2, GZMB, CCL4, IFITM3, RBM25, GZMH, and NKG7 was induced by MWA in GZMH^+^CD8^+^T cells.

CD8^+^T cells strongly expressed CST7, GZMA, GZMB, IFN‐gamma, NKG7, and PRF1, related to cytotoxic function. To determine the immune response of MWA, the biological scores^[^ ^19^ ^]^ were evaluated before and after ablation. The naïve score of CD8^+^ T cells after MWA was significantly lower than that before MWA, and the activation score was significantly increased (Figure 3B), mainly in GZMH^+^CD8^+^T cells (Figure S2, Supporting Information). Importantly, the cytotoxic function of CD8^+^ T cells was significantly higher than that before MWA, in both CD8^+^ T cell clusters (Figure 3C). These results suggested that MWA of early‐stage breast cancer enhanced the cytotoxic activity and chemokine activity of peripheral CD8^+^ T cells.

### 2.5 Increased Inhibitory Signature of Activated CD8^+^ T Cells after MWA

The coinhibitory molecule PD‐1 is induced following T‐cell activation with expression maintained via repeated signaling through the T‐cell receptor (TCR). As a consequence, PD‐1 is a marker of tumor‐associated antigen‐specific T cells.^[^ ^20^ ^]^ Therefore, the activated CD8^+^T cells induced by MWA may show inhibitory characteristics. We compared the inhibitory characteristics of CD8^+^T cells after ablation with that before MWA. After ablation, the mRNA levels of several inhibitory gene seemed to increase in both CD8^+^ T cell clusters, especially for PDCD1 (Figure 2C). To further determine the inhibitory phenotype of CD8^+^ T cells, the inhibitory signature^[^ ^19^ ^]^ was calculated before and after MWA, and the signature was very low before and after ablation. After MWA, CD8^+^T cells showed a marginally significantly increased expression of inhibitory signature in comparison to that before MWA, with a significant difference in GZMK^+^CD8^+^T cells (Figure 3D).

Previous studies^[^ ^21^ ^]^ suggest that CD8^+^T cells show continuous progression from an early effector “transitional” into a terminally differentiated, dysfunctional T cell state, and this dysfunctional terminal CD8^+^T cells also show high exhausted markers and impaired secretion function of IFN‐*γ*. In this study, we found that the average mRNA expression levels of IFNG in both CD8^+^T cell clusters increased after MWA (Figure 2C), and the percentage of IFNG^+^CD8^+^T cells in CD8^+^T cells after MWA was not significantly different from that before ablation (Figure 3E). Above results suggested that peripheral CD8^+^ T cells, activated by MWA of breast cancer, showed increased inhibitory signature but not more dysfunctional, and the increased inhibitory signature did not represent the exhausted phenotype.

### 2.6 Enhanced Co‐Stimulatory Signature of Peripheral CD4^+^ T Cells after MWA

CD4^+^T cells are required for adaptive immune response. Previous studies^[^ ^5^, ^9^, ^14^ ^]^ suggest that early CD4^+^ T cell activation is induced by thermal ablation. To fully investigate the MWA‐induced CD4^+^T cell response, three CD4^+^ T cell clusters were identified. Cells of the first CD4^+^ cluster, CD4_c1, specifically expressed naïve marker genes such as CCR7 and LEF1. The second cluster, CD4_c2, was characterized by the high expression of co‐stimulatory molecules ICOS and CD82 in the early phase of T cell activation, and BIRC3 which inhibits apoptosis, named as CD82^+^CD4^+^T cells, also named as LTB^+^CD4^+^T cells in the previous study.^[^ ^22^ ^]^ The third cluster, CD4_c3, with high expression of CCR7 and TCF7, specifically expressed RCAN3 and CAMK4, both of which are involved in calcium‐dependent signaling pathways and may be important in T cell co‐stimulation. After MWA, patients had increased proportions of CD82^+^CD4^+^T cell (CD4_c2), without significant difference (Figure 2B).

Early T cell activation requires an antigen‐specific signal mediated by the T cell receptor (TCR) plus additional co‐stimulatory signals. Co‐stimulatory genes ICOS, SLAMF1 and TNFRSF25 were expressed in CD4^+^ T cells and the levels of these genes were increased after MWA (Figure 2C). To further investigate the transcriptional states of CD4^+^ T cells after MWA, the expression scores^[^ ^19^ ^]^ of regulatory, co‐stimulatory, and inhibitory T cell phenotypes were determined. The expression of regulatory and inhibitory signatures did not significantly change after MWA (Figure S3, Supporting Information). Interestingly, CD4^+^T cells in peripheral blood after MWA showed increased expression of co‐stimulatory signatures in comparison to that before MWA, mainly in the first and second CD4^+^ T cell clusters (CD82^+^CD4^+^T cell, CCR7^+^CD4^+^T cell) (Figure 3F). The negative co‐stimulatory molecule CTLA‐4 was high expressed in CD82^+^CD4^+^T cells, and the expression level was decreased after MWA (Figure 2C). These results suggested that MWA of breast cancer induced increased co‐stimulatory signatures of CD4^+^T cells, especially for CD82^+^CD4^+^T cells.

### 2.7 Clonal Peripheral T‐Cell Expansion after MWA

**Figure 4:**
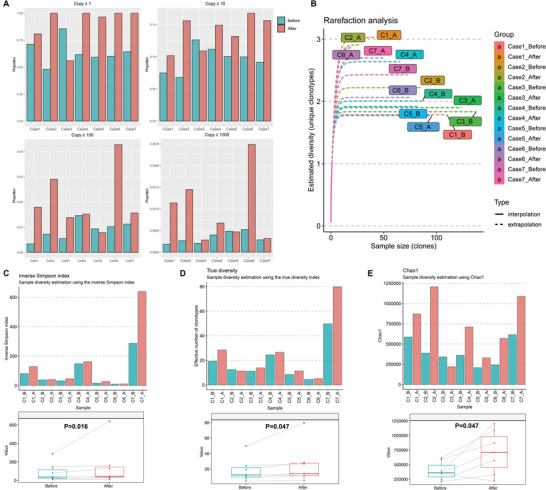
T‐cell clone expansion and TCR repertoire diversity induced by MWA of breast cancer. A) Effect of MWA on clonal expansion. Each graph depicts the proportion of peripheral T‐cell clones expanding by the specified threshold before and after ablation (*n* = 7). B) Estimated diversity before and after MWA of these 7 cases by using rarefaction analysis. The differences of TCR repertoire diversity before and after MWA by defining three indicators (*n* = 7), including C) inverse Simpson index, D) the true diversity index, and E) Chao1 index.

MWA may create a tumor antigen source, and the peripheral TCR diversity may increase after MWA of breast cancer. Ultradeep sequencing approach was applied to characterize T cell receptor *β* repertoires using RNAs isolated from peripheral T cells of consecutive 7 patients before and after MWA. The pre‐/post‐treatment change in abundance of each individual T‐cell clone in peripheral blood was calculated. Of these 7 cases, the percentage of T‐cell clones expanded after MWA in 6 cases with the threshold set to ≥1 copy (**Figure** **4**A), and the similar result was observed with the threshold set to 10 (Figure 4A). At higher thresholds (10^2^ or 10^3^), the expansion of T‐cell clones was also found in these cases (Figure 4A).

Then, we compared the peripheral TCR diversity before ablation to that after MWA of breast cancer. Of these 7 cases, estimated diversity after MWA was higher than that before MWA in 6 cases by using rarefaction analysis (Figure 4B). Moreover, the TCR repertoire diversity was determined by defining the three commonly used indicators, including inverse Simpson index, the true diversity index and Chao1 index. TCR diversities after MWA were significantly higher compared to that before MWA by using above three indexes (Figure 4C,D,E).

### 2.8 Weakly Enhanced Antigen‐Presenting Activity in Myeloid Cell Subsets

In situ tumor ablation can create an antigen source for the generation of antitumor immunity, and these antigens were presented to T cells by APCs, particularly professional APCs, including DCs and macrophages. To determine which type of APCs play the central role in MWA‐induced immune response, we determined the changes of the transcriptional characteristics of peripheral monocytes and DCs.

We re‐clustered myeloid cells and identified 12 distinct cell types (Figure S4A, Supporting Information): three classical monocyte clusters (M1‐3), two nonclassical monocyte clusters (M4, M5), platelets (M6), DC (M7), pDC (M8), basophils (M9), and three neutrophil clusters (M10, M11, M12). Compared with that before MWA, all monocyte clusters showed decreased proportions, without significant difference, and both DC clusters showed stable proportions after MWA (Figure S4B, Supporting Information).

Neutrophils are the predominant leucocytes in the blood and act as the first line of host defense against pathogens, and neutrophils were activated by MWA in this study (data not shown). Gene set variation analysis found that monocytes and DC were enriched in genes associated with adaptive immune response pathway (Figure S4C, Supporting Information). Of these monocyte and DC clusters, no antigen‐presenting related pathway was activated by MWA of breast cancer. Only one major histocompatibility complex (MHC)‐II molecule, HLA‐DRB5, was significantly increased after MWA in four monocyte clusters (M1, M2, M4, and M5) and DC, may indicating weakly enhanced activity of MHC‐II antigen‐presentation induced by MWA (Figure S4D, Supporting Information).

### 2.9 Activated Antigen Receptor‐Mediated Signaling Pathway in B Cell Subsets

Above results found that the antigen‐presenting function of DC and monocytes did not significantly changes after MWA. B cells, in addition to their function in antibody production, can present antigens to CD4^+^T cells.^[^ ^23^ ^]^ We hypothesized that B cells may be dominant APCs initiating CD4^+^ T cell response induced by MWA.

**Figure 5:**
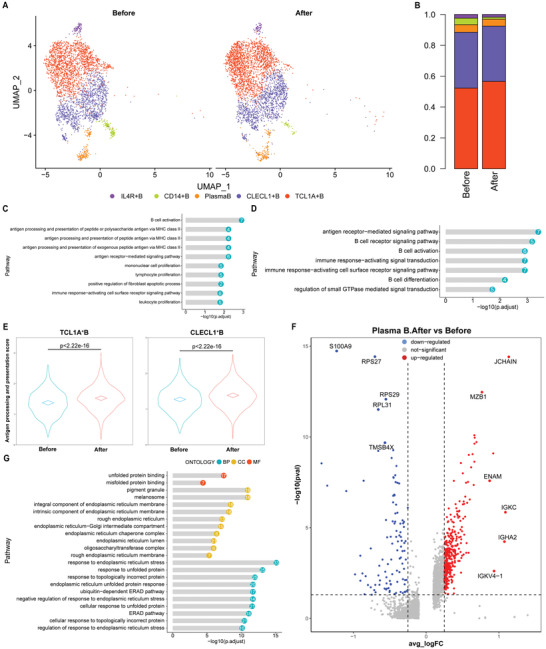
Activated B cells after MWA of breast cancer, with activated antigen receptor‐mediated signaling pathway (*n* = 6). A) UMAP analysis of peripheral B cells showing 5 clusters before and after MWA. B) The proportions of each B cell cluster before and after ablation. GO enrichment pathway analysis of genes preferentially upregulated in C) TCL1A^+^B cells and D) CLECL1^+^B cells after MWA in comparison to that before MWA. E) Antigen processing and presentation scores of TCL1A^+^B cells and CLECL1^+^B cells before and after ablation. F) Volcano plot showing upregulated genes of peripheral plasma B cells induced by MWA of breast cancer. G) GO enrichment analysis showing upregulated pathways in plasma B cells induced by MWA of primary breast cancer.

By projecting the gene expression data of B cells using diffusion maps, we identified five B cell clusters using scRNA‐seq (**Figure** **5**A and Figure S5A, Supporting Information): TCL1A^+^B cells (B1); CLECL1^+^B cells (B2); plasma B cells (B3); CD14^+^B cells (B4); and IL4R^+^B cells (B5).^[^ ^19^, ^24^ ^]^ TCL1A^+^B cells specifically expressed TCL1A, IGHD, IL4R, PLPP5, FCER2, CXCR4, APLP2, and HVCN1, indicative of high activity of antigen presentation. CLECL1^+^B cells expressed CLECL1, SSPN, AIM2, GPR183, TNFRSF13B, ITGB1, TFEC, and CD82. Plasma B cells specifically expressed IGHA1, JCHAIN, IGHG1, IGHA2, IGHG4, IGHG3, IGHG2, IGKC, IGHGP, and MZB1. CD14^+^B cells specifically expressed inflammatory genes MNDA, AIF1, TYROBP, and CD14. IL4R^+^B cells expressed TAOK1, SPATA22, CMC2, IFT57, CD63, and RBBP7.

In comparison with that before MWA, the proportion of each B cell cluster remained stable except for CD14^+^B cell after MWA, with a decreased proportion >50% (Figure 5B and Figure S5B, Supporting Information). GO enrichment analysis indicated that TCL1A^+^B cells (B1) and CLECL1^+^B cells (B2) after MWA were specifically enriched in genes associated with B cell activation pathway, antigen receptor‐mediated signaling pathway, and immune response‐activating cell surface receptor signaling pathway compared with that before ablation (Figure 5C,D). Moreover, the genes associated with lymphocyte proliferation, mononuclear cell proliferation, leukocyte proliferation, and lymphocyte activation pathways were enriched in B1 cluster after MWA in comparison to that before MWA (Figure 5C).

Furthermore, significantly increased expression of MHC‐II (HLA‐DQA2, HLA‐DRB6, HLA‐DQA1 and HLA‐DMB in B1 cluster; HLA‐DRB1 and HLA‐DRB6 in B2 cluster) was observed after MWA (Figure S5C, Supporting Information). Then antigen processing and presentation score was computed.^[^ ^25^ ^]^ The scores of TCL1A^+^B cells and CLECL1^+^B cells were significantly increased after ablation in comparison to that before MWA (Figure 5E), indicating the increased the ability of MHC‐II antigen‐presentation induced by MWA in B1 and B2 cluster but not in other B cell clusters.

Plasma B cells in peripheral blood after MWA showed increased expression of genes associated with anti‐body production, such as JCHAIN, IGKC, IGHA2, IGKV4‐1, ENAM, and MZB1 in comparison to that before MWA (Figure 5F). Moreover, GO enrichment analysis (Figure 5G) indicated plasma B cells after MWA were enriched in genes associated response to unfolded protein pathway, response to topologically incorrect protein pathway, endoplasmic reticulum unfolded protein response pathway, and cellular response to unfolded protein pathway, suggesting that plasma B cells showed increased ability of anti‐body production induced by MWA. All these results suggested that B cells were activated by MWA of breast cancer, with activated antigen receptor‐mediated signaling pathway.

### 2.10 Cell–Cell Communication Contributed to CD4^+^ T Cell Immune Response

**Figure 6:**
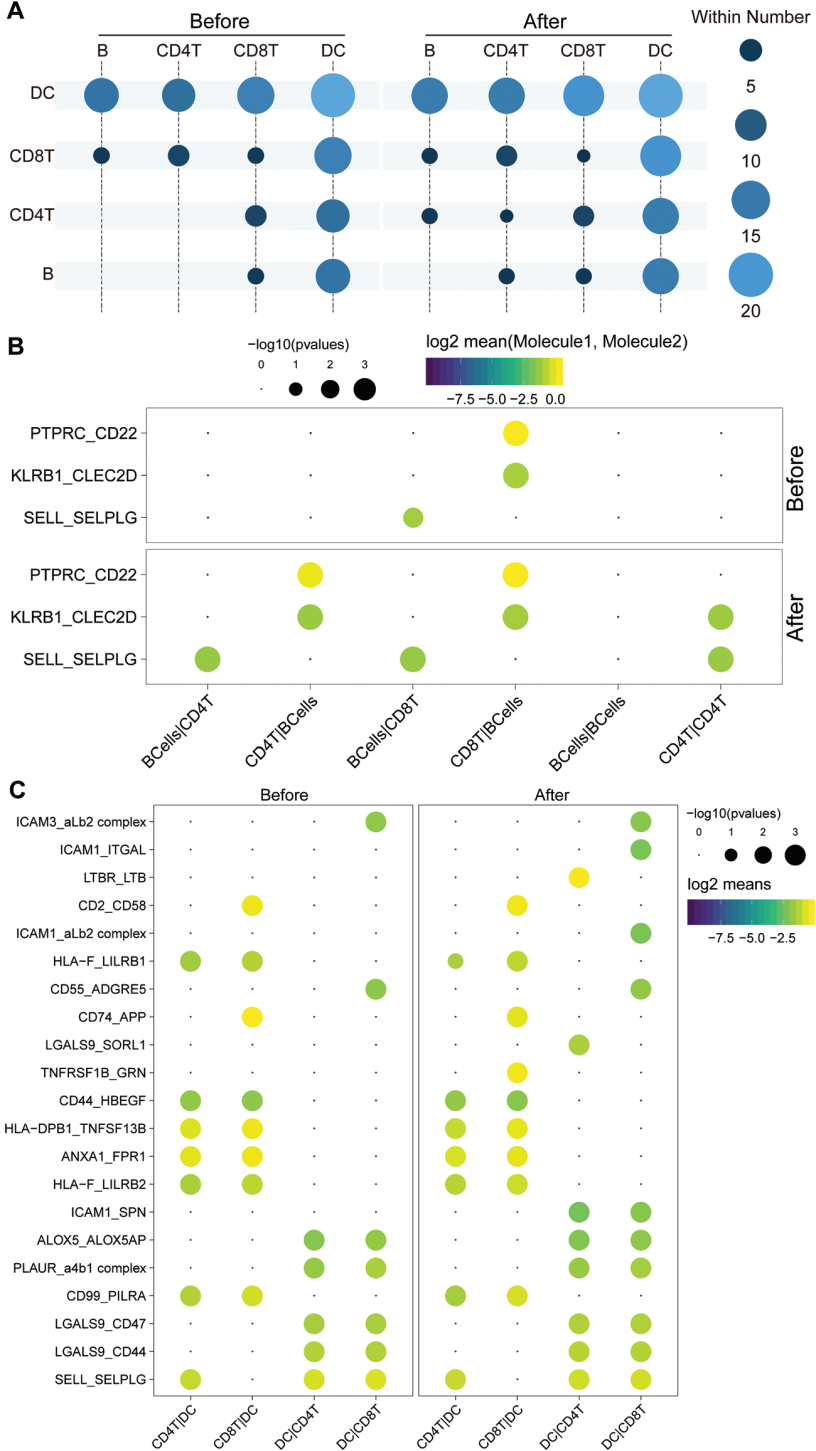
Cell‐to‐cell communications between antigen‐presenting cells (APCs) and T cells in the peripheral blood before and after MWA in the treatment of breast cancer (*n* = 6). A) The numbers of cell–cell interactions between APCs and T cells. B) Overview of selected ligand–receptor interactions between B cells and CD4^+^ or CD8^+^ T cells before and after MWA. C) Overview of selected ligand–receptor interactions between DC and CD4^+^ or CD8^+^ T cells before and after MWA. *P* values indicated by circle size (permutation test). The means of the average expression level of interactions are indicated by color.

To predict cell–cell interactions that may contribute to the MWA‐induced immune response, CellphoneDB was applied.^[^ ^26^ ^]^ First, we calculated the interactions between cell types both before and after MWA. Interestingly, we observed more interactions between CD4^+^ T cells and B cells or CD4^+^ T cells in the peripheral blood after MWA in comparison to that before MWA (**Figure** **6**A). Specifically, enhanced adhesion interaction SELL‐SELPLG and cytotoxic interaction KLRB1‐CLEC2D between CD4^+^ T cells and B cells or CD4^+^ T cells were observed after MWA than that before MWA (Figure 6B). Importantly, enhanced interaction PTPRC‐CD22, associated with antigen receptor signaling, between CD4^+^ T cells and B cells was found induced by MWA (Figure 6B).

DCs are the most potent professional APCs, presenting antigen in the context of MHC class I and II molecules to CD8^+^ and CD4^+^ T cells. Here, we found more interactions between DCs and T cells in the peripheral blood after MWA than that before MWA (Figure 6A). Specifically, enhanced interactions LTBR‐LTB, LGALS9‐SORL1, and ICAM1‐SPN between DCs and CD4^+^ T cells in the peripheral blood induced by MWA were observed (Figure 6C). Moreover, enhanced interactions TNFRSF1B‐GRN, ICAM1‐ITGAL, ICAM1‐aLb2 complex, and ICAM1‐SPN between DCs and CD8^+^ T cells were found after MWA (Figure 6C).

Above all, we found that enhanced interactions between B cells and CD4^+^ T cells were induced by MWA, which was validated by another method CellChat (Figure S6, Supporting Information), indicating that B cells were important APCs that initiate CD4^+^T cells in MWA‐induced immune response.

### 2.11 Validations of Global Immune Response Characterization

**Figure 7:**
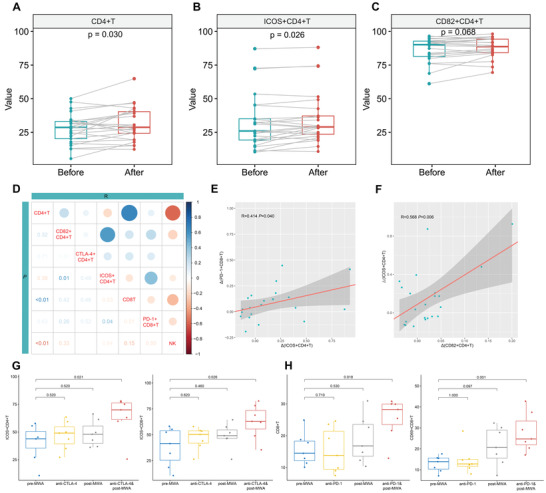
Validations of MWA‐induced immune response and combination experiments in vitro. A–C) The proportions of peripheral CD4^+^, ICOS^+^CD4^+^, and CD82^+^CD4^+^ T cells after MWA, higher than that before MWA (*n* = 25). D) The correlations of the MWA‐induced changes of immune cells between different subtypes (*n* = 25). The upper right part represents the correlation *R* value between indicators, and the lower left part represents their *p* values. The darker the color presented the higher *R* value or the lower *p* value. Red means the negative correlation, and blue means positive. E) Correlations of MWA‐induced increased proportion between ICOS^+^CD4^+^ T cells and PD‐1^+^CD8^+^ T cells, and F) CD82^+^CD4^+^ T cells and ICOS^+^CD4^+^ T cells (*n* = 25). G) Blockade of CTLA‐4 for PBMCs after MWA showing increased proportions of ICOS^+^CD4^+^ T cells and ICOS^+^CD8^+^ T cells in comparison to that in PBMCs before MWA (*n* = 7). H) PD‐1 inhibitor cultured PBMCs after MWA showing significantly higher levels of CD8^+^ and CD69^+^CD8^+^ T cells than that in pre‐MWA PBMCs (*n* = 7).

To confirm the scRNA‐seq results, the peripheral T and NK cell subsets were determined by flow cytometric analysis of PBMCs from 25 patients (Table S2, Supporting Information). The peripheral frequencies of CD8^+^ T cells and NK cells after MWA were not significantly higher than those before MWA, and the frequency of CD4^+^ T cells significantly increased after MWA (*p* = 0.030, **Figure** **7**A). Then, the co‐stimulatory CD4^+^ T cell subsets were further determined (Figure 7B,C). The frequency of CD82^+^CD4^+^ T cells increased after MWA with a marginally significant difference (*p* = 0.068). Importantly, the peripheral frequency of ICOS^+^CD4^+^ T cells after MWA was significantly higher than that before MWA (*p* = 0.026). PD‐1 is a marker of tumor‐associated antigen‐specific T cells.^[^ ^20^ ^]^ The frequency of PD‐1^+^CD8^+^ T cells showed a trend to increase after MWA, without a significant difference (data not shown).

As we know, different subtypes of immune cells synergistically form antitumor immune response. According to above results, the relationships among different immune cells were determined (Figure 7D). The increased level of CD4^+^ T cells was positively associated with that of CD8^+^ T cells, but negatively associated with that of NK cells. Moreover, the increased level of ICOS^+^CD4^+^ T cells was positively associated with that of PD‐1^+^CD8^+^ T cells (Figure 7E), suggesting that CD4^+^ and CD8^+^ T cell immune response may be associated with MWA‐induced antigens release. Interestingly, the increased level of CD82^+^CD4^+^ T cells was positively related to that of ICOS^+^CD4^+^ T cells (Figure 7F), indicating that CD82 may be another co‐stimulatory marker of CD4^+^ T cells.

Because of the limited sample size, subgroup analysis was not performed. Enhanced proportion of ICOS^+^CD4^+^ T cells or CD8^+^ T cells after MWA was observed in both ER positive and negative cases, HER2 positive and negative cases, young and old patients. Interestingly, combination analyses of cases 115 and 117 found that the cytotoxic function of NK and CD8^+^ T cells was still enhanced after MWA (Figure S7, Supporting Information), although both cases showed decreased proportions of NK and T cells. Moreover, enhanced co‐stimulatory signature of peripheral CD4^+^ T cells after MWA was still found from combination analysis of cases 115 and 117 (Figure S7, Supporting Information).

### 2.12 Synergistically Activated T Cells after Ablation by Immune Checkpoint Inhibitors In Vitro

The costimulatory signature of CD4^+^ T cells was significantly increased induced by MWA, and CTLA‐4 was highly expressed in peripheral CD4^+^ T cells in this study. CTLA‐4 blockade can provide additional signal to costimulatory pathway.^[^ ^27^ ^]^ PBMCs from 7 patients (Table S2, Supporting Information) before and after MWA were obtained for further experiments. Because ICOS can actively participate in enhancing immune responses against tumors,^[^ ^28^ ^]^ the ICOS expression level of CD4^+^ and CD8^+^ T cells was tested to determine the combination effect of MWA and CTLA‐4 inhibitor. Our ex vivo study showed that the proportion of ICOS^+^CD4^+^ T cells in KN044 (CTLA‐4 inhibitor) cultured PBMCs after MWA was significantly higher than that in PBMCs before MWA (*p* = 0.021, Figure 7G). Moreover, this group showed a highest proportion of ICOS^+^CD8^+^ T cells, with a significant difference in comparison to PBMCs before MWA (*p* = 0.026, Figure 7G).

Moreover, the inhibitory signature, including PD‐1 and other exhausted genes, of activated CD8^+^ T cells was increased induced by MWA of breast cancer. As a coinhibitory molecule, blockade of PD‐1 leads to reinvigoration of T cell function and effective antitumor responses. We found that camrelizumab (PD‐1 inhibitor) cultured PBMCs after MWA showed a higher proportion of CD8^+^T cells compared with PBMCs before MWA (*p* = 0.018, Figure 7H). Importantly, a significantly higher percentage of CD69^+^ activated CD8^+^T cells was observed in camrelizumab cultured PBMCs after MWA in comparison to PBMCs before MWA (*p* = 0.001, Figure 7H). Above results suggested that peripheral T cells after MWA were synergistically activated by immune checkpoint inhibitors in vitro.

## 3 Discussion

In recent years, immunotherapy has emerged as a novel option for several tumors, including breast cancer. For the immunologically cold breast cancer, combinatorial strategy is urgently needed to improve the effect of immunotherapy. Minimally invasive therapies have been attempted in the treatment of early‐stage breast cancer. As an effective local therapy, minimally invasive thermal therapy may be a trigger of antitumor immunity, and its combination with immunotherapy may be a promising strategy in the treatment of early‐stage breast cancer. However, the immune response induced by minimally invasive therapies has not been fully reported. To the best of our knowledge, global characteristics of MWA‐induced systemic immune response was firstly reported;^[^ ^11^, ^14^ ^]^ B cells were important APCs involved in MWA‐induced immune response; MWA combined with immune checkpoint inhibitor showed enhanced immune response.

For early‐stage breast cancer, most cases are cured by resection of the primary tumor combined with systemic therapy. However, some patients still relapsed, which may attribute to circulating tumor cells and post‐treatment immunosuppressive microenvironments.^[^ ^17^ ^]^ Surgery may disseminate cancer cells and accelerate residual tumor growth. Interestingly, we found peripheral cytotoxic T cells and NK cells were activated by MWA of breast cancer, although the characteristics of all enrolled patients were heterogeneous. MWA is not only an effective local therapy, but also a trigger of antitumor immunity for early‐stage breast cancer, may leading to better survival than surgery. Because antitumor immunity after MWA of breast cancer reported by previous studies and our data was weak,^[^ ^11^, ^14^ ^]^ future clinical studies are needed to confirm the long‐term outcome of MWA for the treatment of early‐stage breast cancer. Previous studies^[^ ^3^, ^4^, ^5^ ^]^ have shown that the efficacy of immunotherapy is promising in TNBC, but not other subtypes, suggesting that TNBC is immunogenic. Similarly, the previous study^[^ ^14^ ^]^ has found that MWA‐induced Th1‐type immune response mainly in non‐luminal breast cancers. Interestingly, MWA‐induced antitumor immune response was also observed in several luminal cases in this study and the previous study.^[^ ^14^ ^]^ Moreover, the preclinical studies^[^ ^29^ ^]^ have shown that incomplete ablation induced immunosuppression after thermal ablation, and decreased proportion of NK and T cells was observed in one case without complete ablation. In our clinical practice, incomplete ablation was seldom observed, and whether incomplete ablation is associated with immunosuppression should be determined. Besides, the peripheral immune cell landscape is reprogrammed with age,^[^ ^30^ ^]^ and age may be associated with MWA‐induced immune response. Importantly, NK and T cells were still activated by MWA in cases 115 and 117 with decreased proportions of NK and T cells, indicating that decreased proportions did not mean decreased functions of these cells. Future biomarker studies are needed to determine who will be benefit from MWA with enhanced systemic anti‐tumor immune response.

The underlying mechanisms and key immune cell subsets of thermal therapy induced systemic immune response are not clear. In this study, we found that systemic NK and CD8^+^ T cells were activated with enhanced the cytotoxic activity and chemokine activity, but the absolute number of these cells did not significantly increased. The thermal therapy induced weak systemic immune response has also been reported by previous studies.^[^ ^6^, ^9^, ^10^, ^14^ ^]^ Moreover, the expansion of T‐cell clones was observed confirmed by TCR sequencing of peripheral T cells, suggesting antigens release induced by MWA. DCs are the most potent professional APCs, but antigen‐presenting related pathways of peripheral monocytes and DCs were not activated after MWA. The previous study^[^ ^11^ ^]^ has found infiltrated DCs and macrophages were significantly increased after ablation. We inferred that DCs and macrophages may play an important role in antigen‐presenting in situ but not in the peripheral blood. Interestingly, we firstly reported that the antigen‐presentation function of peripheral B cells was significantly enhanced after MWA of breast cancer, to the best of our knowledge. More interaction between B cells and CD4^+^ T cells were found after MWA in comparison to that before ablation. Neoantigen‐driven B cell and CD4^+^ T cell collaboration promotes anti‐tumor CD8 T cell responses.^[^ ^31^ ^]^ Our results suggested B cell and CD4^+^ T cell collaboration may play an important role in MWA‐induced systemic immune response.

Due to the low response rate of single immunotherapy for breast cancer, several combinatorial strategies are attempted.^[^ ^4c^ ^]^ Chemotherapy combined with immunotherapy has shown favorable effect for advanced breast cancer, including nab‐paclitaxel, adriamycin, and carboplatin. However, chemotherapy‐related adverse events cannot be ignored.^[^ ^4^ ^]^ Our data provided global characteristics of systemic anti‐tumor immune response induced by MWA in the treatment of breast cancer, and several potential targets were found. According to our data, MWA combined with CTLA‐4 inhibitor and PD‐1 inhibitor showed enhanced T cell response confirmed by ex vivo experiments. Compared to chemotherapy, no systemic adverse effects have been reported by MWA. This combinational strategy may be promising for the treatment of early‐stage breast cancer, with favorable local and systemic effects. Several clinical trials (NCT03546686, NCT04805736) are ongoing to test the effect of thermal ablation combined with immunotherapy.

Several limitations still existed. First, this is a window‐of‐opportunity study, and surgery was performed to these patients within two weeks after ablation. Only short‐term immune response was determined, and long‐term immune response should be investigated in the future. Second, APCs are constantly sampling the local environment for antigens after MWA of the primary breast cancer. Local antigen processing and presentation is the initial step of MWA‐induced immune response. However, the characteristics of this initial immune process was not investigated due to the difficulty to obtain the drainage lymph nodes. Third, because of the limited sample size, our findings were generated independent from different clinicopathological factors and different local control, and future studies with large sample sizes are needed to find the potential patients who will be benefit from this local therapy with additional enhanced immune response. Fourth, the number of cell–cell interactions in the peripheral blood was relatively small. Although the results of these interactions by using different methods were different, enhanced interactions between B cells and CD4^+^ T cells were observed by using two methods in this study. Fifth, immune checkpoint inhibitors activated T cells post‐MWA in vitro; however, the anti‐tumor activity of MWA combined with immune checkpoint inhibitors is still not clear. Future clinical trials are needed to investigate the effect of this combination strategy.

## 4 Conclusions

This study provided global characteristics of MWA‐induced systemic immune response, and the corresponding molecular features were also reported. Besides, B cells were important APCs involved in MWA‐induced immune response. Importantly, the current study paved a way for the identification of potential targets to improve the immune response of MWA for breast cancer.

## 5 Experimental Section

### Patients and Study Design

A clinical trial (ChiCTR2000029155) was performed to determine the short‐term local effect of MWA in the treatment of breast cancer, and approved by the institutional ethics committee of the hospital. Informed consent was obtained from all enrolled patients. All experiments were in accord with the Helsinki Declaration. 29 patients were enrolled in this study, and the peripheral blood was withdrawn on the day before and 1 week after MWA for further experiments according to the previous studies^[^ ^5^, ^13^, ^14^ ^]^ to determine the potential immune response induced by MWA. Then, PBMCs were isolated.

The eligibility criteria for the present study included the following: (1) a single tumor without an extensive intraductal component; (2) invasive breast cancer proved by using core‐needle biopsy; (3) breast cancer 3.0 cm or less in greatest diameter confirmed by using ultrasound; (4) female patients, old than 18 years, without coagulative disease, chronic liver disease, renal failure, immune system diseases or other acute and chronic diseases, which may have an effect on the immune response.

For scRNA‐seq, PBMCs before and after MWA from consecutive 6 patients were applied from Feb 2020. To characterize TCR *β* repertoires, ultradeep sequencing approach was applied using RNAs of peripheral T cells of consecutive 7 patients before and after MWA. To confirm the results of scRNA‐seq, the main immune cell subtypes were determined by flow cytometric analysis. PBMCs of 25 cases were available for flow cytometric analysis. Finally, PBMCs of 7 cases were used for ex vivo experiments to test the combination treatment strategy.

### MWA and Other Therapies

In this window‐of‐opportunity study, MWA was performed to enrolled patients diagnosed with breast cancer. Then the prescheduled surgery was performed within two weeks after ablation. Subsequent treatments were recommended according to the guidelines.

Under local anesthesia, MWA was performed according to previous studies.^[^ ^7^, ^14^, ^16^ ^]^ The microwave irradiation frequency of the system (Nanjing Yigao Microwave Electric Institute, Nanjing, China) was 2450 MHz with an output power set at 40 W, and 1–5 min were needed for complete ablation on ultrasound. Complete ablation was defined as no viable tumor cells determined by pathological assessments after surgery.

### Isolation of PBMCs from Patients

PBMCs were isolated by Ficoll discontinuous density gradient centrifugation within 2 h of blood sample collection and frozen in 90% fetal bovine serum (FBS) + 10% DMSO (Sigma, USA) freezing medium for later use.

### Single‐Cell RNA Sequencing of PBMCs

The cellular viability of all freshly PBMCs exceeded 90% evaluated by trypan blue (Sigma, USA) microscopically. Single‐cell suspensions (1 × 10^5^ cells mL^−1^) with PBS (HyClone, USA) were loaded into microfluidic devices using the Singleron Matrix Single Cell Processing System (Singleron, China). Subsequently, the scRNA‐seq libraries were constructed according to the protocol of the GEXSCOPE Single Cell RNA Library Kits (Singleron, China).^[^ ^32^ ^]^ Individual libraries were diluted to 4 × 10^−9^ m and pooled for sequencing. At last, pools were sequenced on Illumina HiSeq X with 150 bp paired end reads.

### Single Cell RNA‐seq Data Processing and Quality Control

Sequencing outputs were demultiplexed to convert BCL files to FASTQ format using bcl2fastq (illumina), and sequencing data were processed by using the CeleScope pipeline (https://gitee.com/singleron‐rd/celescope, Singleron). Adapters and poly A tails were trimmed (fastp V1) before aligning read two to GRCm38 using Ensemble v.92 gene annotation (fastp 2.5.3a and feature Counts 1.6.2). Reads with the same cell barcode, UMI, and gene were grouped together to calculate the number of UMIs per gene per cell. The UMI count tables of each cellular barcode were used for further analysis.

The cells that had either lower than 200 or higher than 5000 expressed genes were removed. Furthermore, cells with more than 30 000 UMIs were discarded. Finally, 80 246 cells were obtained after quality control for the downstream analysis. The median genes ranged from 441 to 779 and the median UMIs ranged from 756 to 1653.

### Dimensionality Reduction, Clustering, Cell‐Type Labeling, and Visualization

Seurat v3.1.2 was used for dimensionality reduction, clustering, and visualization. For each sample dataset, the filtered expression matrix to identify cell subsets was used. The filtered gene expression matrix was normalized using Seurat's NormalizeData function, in which the number of UMIs of each gene was divided by the sum of the total UMIs per cell, multiplied by 10 000, and then transformed to logscale (ln (UMI‐per‐10000+1)). Then FindVariable function was applied to select the top 2000 variable genes and perform principal component analysis (PCA). Harmony v1.0 was applied to integrate samples and perform downstream analysis. Clustering with 25 principal components and resolution 1.2 were performed by graph‐based clustering, visualized using Uniform Manifold Approximation and Projection (UMAP) with Seurat functions RunUMAP. For subclustering of T&NK cells and Myeloid cells, the top 20 principal components were selected with a resolution parameter equal to 0.6. For subclustering of B cells, the top 20 principal components were selected with a resolution parameter equal to 0.3. The cell type identity of each cluster was determined by using the canonical markers from literature references, and the reference database SynEcoSys database (Singleron Biotechnologies). Cell doublets were estimated based on the expression pattern of canonical cell markers. Any cluster enriched with multiple cell type‐specific markers was excluded from downstream analysis.

### Differential Gene Expression Analysis and Enrichment Analysis

Differentially expressed genes in a given cell type compared with all other cell types were determined with the FindAllMarkers function from the Seurat package (Wilcoxon rank sum test, p values adjusted for multiple testing using the Bonferroni correction). Genes that expressed in more than 10% of the cells in both of the compared groups and with an average log (Fold Change) value greater than 0.25 were selected as differentially expressed genes (DEGs). Differentially expressed genes were filtered by fold change >0.25 and *p* value < 0.05. For the differential expressed genes of different cell types, genes with *p* value < 0.05 and logFC > 0.25 were selected for enrichment analysis. The up‐regulation genes and down‐regulation genes were analyzed separately. The GO and KEGG enrichment analyses were performed by clusterProfiler (3.16.1).

### Defining T/NK‐Cell and B‐Cell Signature Scores

T/NK‐cell substrate signature gene sets were derived from previously published work.^[^ ^19^ ^]^ The gene sets of antigen processing and presentation score came from the previous study.^[^ ^25^ ^]^ The score of specific signature gene set was calculated by AUCell (v1.8.0, AUCell is an R package for evaluating gene signatures in single‐cell datasets).^[^ ^33^ ^]^

### Cell–Cell Interaction Analysis

To resolve the cellular communications between different cell clusters of samples, CellphoneDB was implemented to detect the cell interactions on the basis of normalized UMI, respectively. Significant cell interactions (*p* value < 0.05) were used for further analysis. Cytoscape was used to visualize and analyze network graphs of cell interaction. CellChat (version 0.0.2) was also used to analyze the intercellular communication networks from scRNA‐seq data. A CellChat object was created using the R package process. Cell information was added into the meta slot of the object. The ligand–receptor interaction database was set, and the matching receptor inference calculation was performed.

### TCR Sequencing

Total RNA from PBMCs were isolated using the RNeasy Mini kit (Qiagen, Hilden, Germany) and cDNA were synthesized using a PrimeScript RT Master Mix (TaKaRa Shuzo Co, Shiga, Japan). TCR*β* chain sequencing libraries were prepared by using Multiplex PCR, and performed to sequencing on the Illumina HiSeq4000 platform (BGI Tech, Shenzhen, China). Details of TCR*β* sequencing were delineated in the previous studies. Only productive TCR*β* CDR3 sequences are the object of this study.

### Flow Cytometric Analysis of PBMCs

After thawing, PBMCs from patients were processed for immunophenotype analysis. Cells were incubated with Human TruStain FcX (Fc Receptor Blocking Solution, BioLegend) to block the Fc receptors. Then, PBMCs were stained with fluorochrome‐labeled antibodies (BioLegend) against the following surface markers: Alexa Fluor 700 anti‐human CD3 (clone OKT3), FITC anti‐human CD4 (clone PRA‐T4), APC/Cyanine7 anti‐human CD8a (clone RPA‐T8), PE/Cy7 anti‐human/mouse/rat CD278 (ICOS) (clone C398.4A), PE anti‐human CD152 (CTLA‐4) (clone L3D10), APC anti‐human CD82 (clone ASL‐24), Brilliant Violet 875‐anti‐human CD279 (PD‐1) (clone EH), Brilliant Violet 510‐anti‐human CD69 (clone FN50). Zombie Aqua Fixable Viability Kit (BioLegend, San Diego, USA) was added to excluded dead cells. Cells were acquired on the BD FACS Aria II cytometer (BD Biosciences), and data were analyzed using FlowJo 10.4 software.

### In Vitro PBMCs Culture

Freshly PBMCs were stimulated with the T Cell TransAct (titer 1:100) (Miltenyi Biotec, Germany) in RMPI 1640 medium (Hyclone, USA) supplemented with 10% FBS, 1% streptomycin, 1% penicillin (Sigma, USA) and 20 IU mL^−1^ human IL‐2 (20 IU mL^−1^ Peprotech, USA) at 37 °C with 5% CO_2_. After stimulating 48 h, the activated PBMCs were incubated in the presence of 20 ug mL^−1^ KN044 (CTLA‐4 inhibitor) (Alphamab, China), 20 ug mL^−1^ Camrelizumab (AiRuiKa) (PD‐1 inhibitor) (Jiangsu Hengrui Medicine, China) for next 72 h. Following incubation, PBMCs were collected and used for flow cytometric analysis.

### Statistical Analysis

Continuous variables with normal distribution or with abnormal distribution were described as mean and standard deviation (SD) or medians and interquartile ranges (IQR), respectively. Categorical variables were described with frequencies and proportions. Paired or unpaired Student's t test and Wilcoxon rank sum test was used for comparisons of continuous variables between two groups as appropriate. Chi‐square test or Fisher's exact test was used to compare these categorical variables. Correlation analysis and R was used to estimate the relationship between two continuous variables. All statistical analyses were performed using R version 4.1.1 (https://www.r‐project.org/). A two‐sided *p* value < 0.05 was considered as statistically significant.

## Conflict of Interest

The authors declare no conflict of interest.

## Author Contributions

W.Z., M.Y., X.M., and H.P. contributed equally to this work. W.Z., S.W., K.Z., W.Q., and Q.D. contributed to the conception and design of the study, the analysis and interpretation of data, the revision of the article as well as final approval of the version to be submitted. W.Z., M.Y., X.M., H.P., W.Q., X.T., J.W., N.C., and K.Z. performed the experimental study and the statistical analysis, drafted and revised the article. H.X., L.L., Y.Z., C.W., Q.D., and X.L. participated in the clinical study, performed the statistical analysis, drafted and revised the article. All authors read and approved the final version of the manuscript.

## Supporting information

Supporting Information

## Data Availability

The data that support the findings of this study are available from the corresponding author upon reasonable request.
