# Supplementary material for: Landscape of the Peripheral Immune Response Induced by Local Microwave Ablation in Patients with Breast Cancer
Source: Adv Sci (Weinh). 2022 Apr 11;9(17):2200033. doi: 10.1002/advs.202200033 (PMC9189675; doi:10.1002/advs.202200033)
Supplement: Supplementary file 1 — Supporting Information [file ADVS-9-2200033-s001.pdf]

ADVANCED SCIENCE
Open Access

Supporting Information

for *Adv. Sci.*, DOI 10.1002/advs.202200033

Landscape of the Peripheral Immune Response Induced by Local Microwave Ablation in Patients with Breast Cancer

Wenbin Zhou, Muxin Yu, Xinrui Mao, Hong Pan, Xinyu Tang, Ji Wang, Nan Che, Hui Xie, Lijun Ling, Yi Zhao, Xiaoan Liu, Cong Wang, Kai Zhang*, Wen Qiu*, Qiang Ding* and Shui Wang**

Landscape of the peripheral immune response induced by local microwave ablation in patients with breast cancer

Supplementary Materials

Figure. S1. The heatmap of gene expression in NK and T cell clusters.

Figure. S2. The upregulated activation score of GZMH+CD8+T cell cluster induced by MWA of breast cancer.

Figure. S3. The regulatory and inhibitory scores in CD4+T cells before and after MWA.

Figure. S4. Changes of the peripheral myeloid cells induced by MWA of breast cancer.

Figure. S5. Changes of the B cells induced by MWA of breast cancer.

Figure. S6. The cell-to-cell interactions among different cell types by using CellChat before and after microwave ablation in the treatment of breast cancer.

Figure. S7. The biological scores before and after MWA from combination analysis of case 115 and 117.

Table. S1. The percentages of different immune cells before and after MWA

Table. S2. Baseline characteristics of study population.

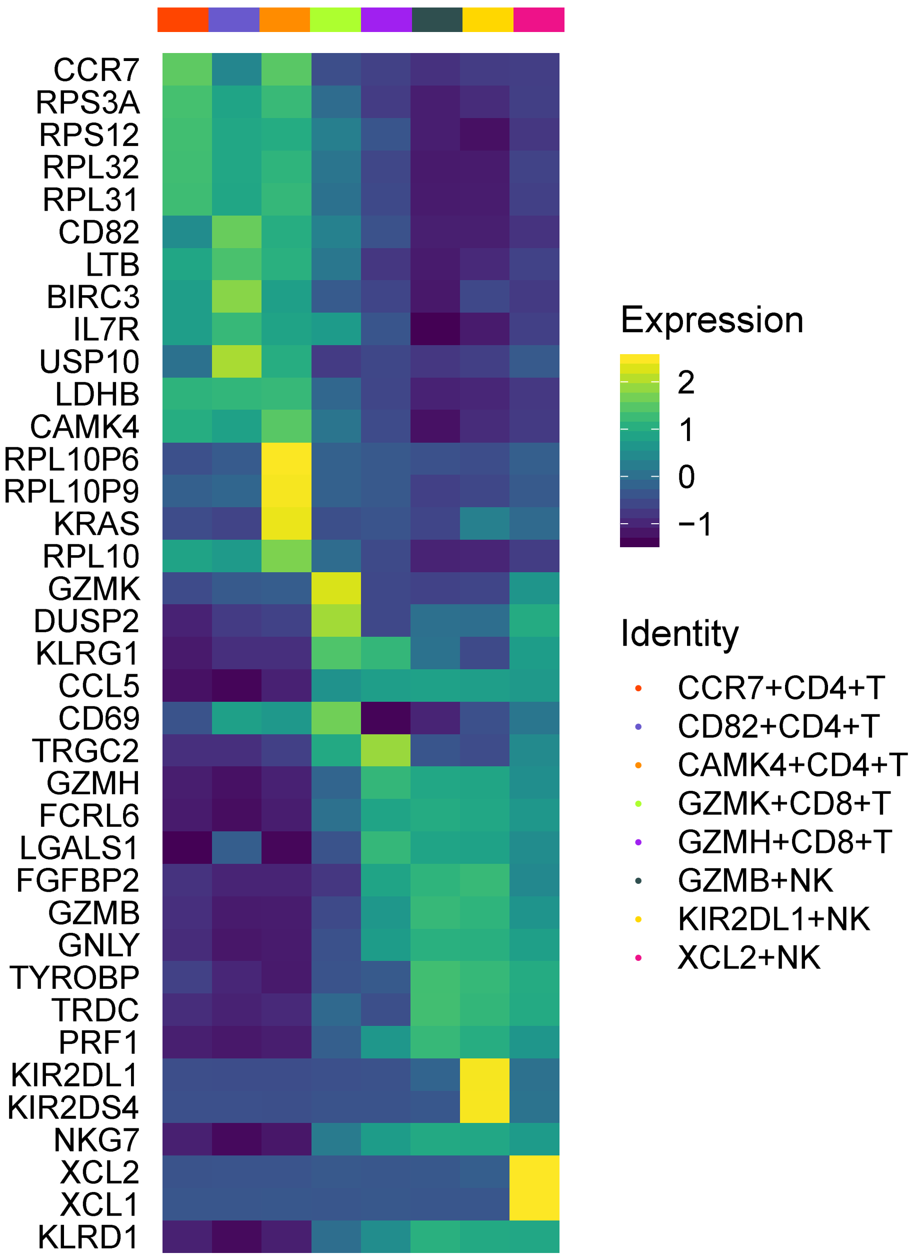

Figure. S1. The heatmap of gene expression in NK and T cell clusters.

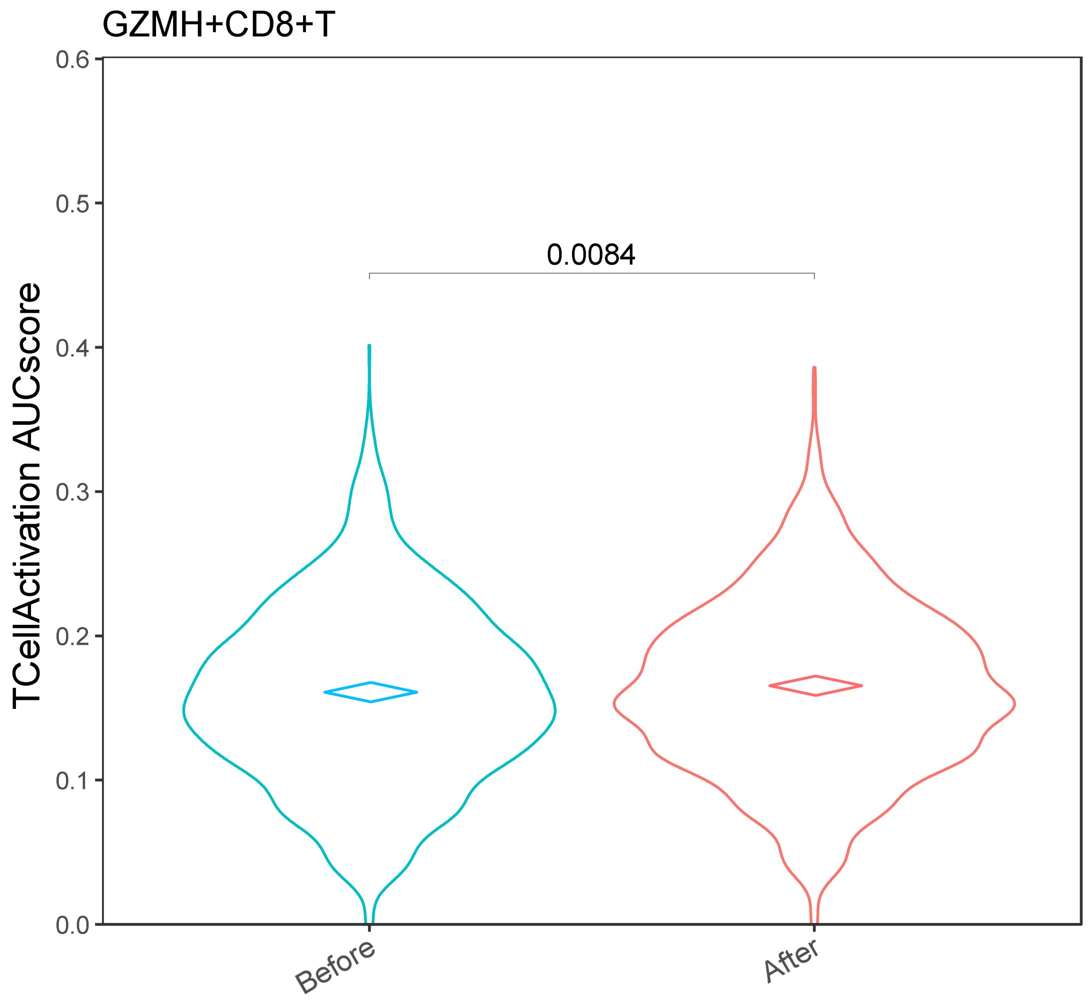

Figure. S2. The upregulated activation score of GZMH+CD8+T cell cluster induced by MWA of breast cancer.

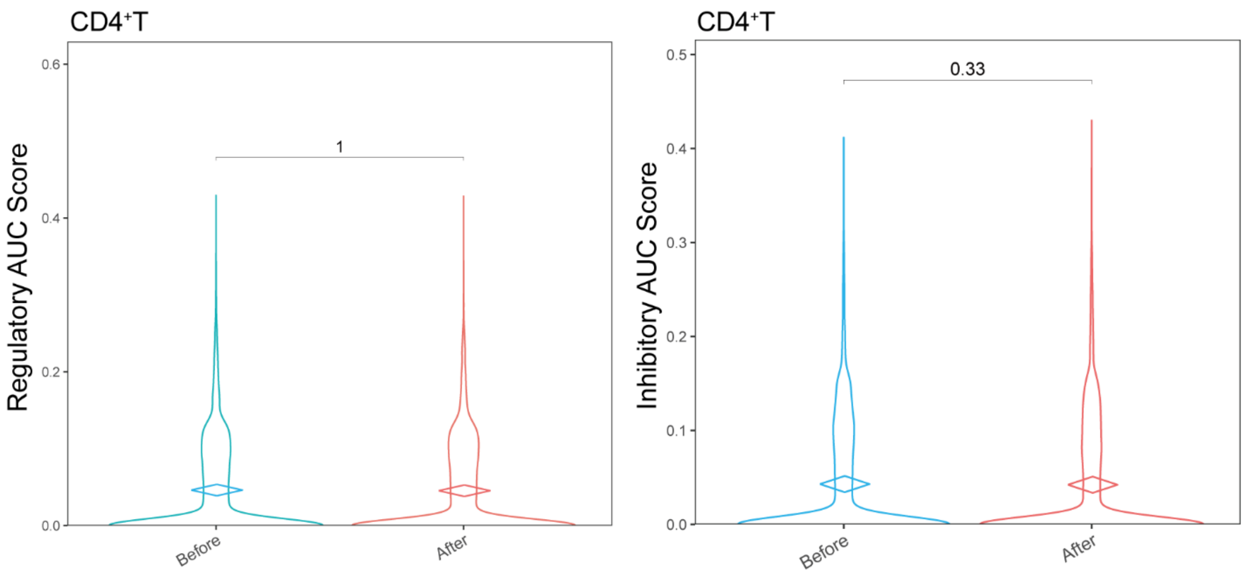

Figure. S3. The regulatory and inhibitory scores in CD4+T cells before and after MWA.

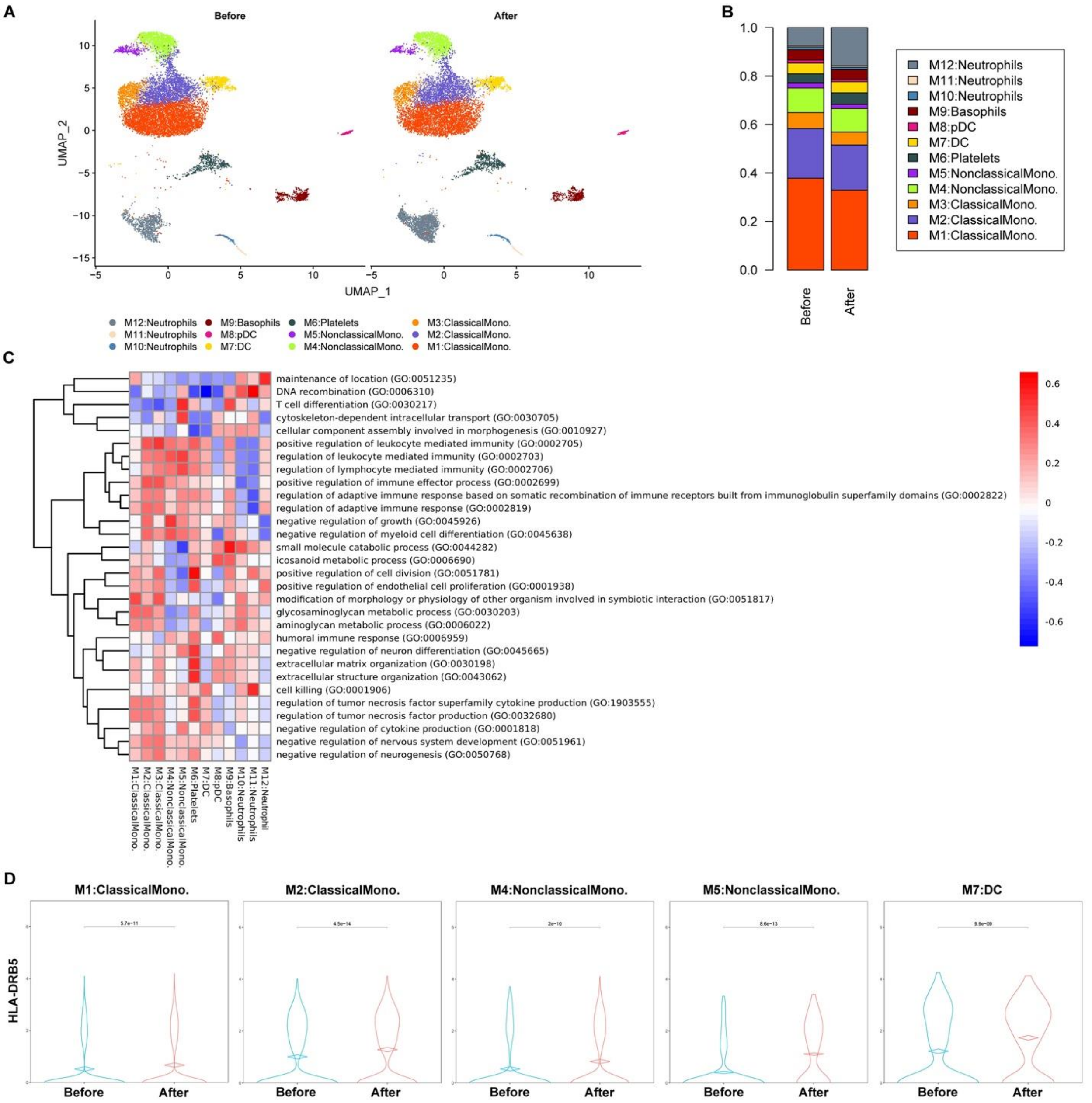

Figure. S4. Changes of the peripheral myeloid cells induced by MWA of breast cancer. (A) UMAP analysis of peripheral myeloid cells showing 12 clusters before and after MWA. (B) The proportions of each myeloid cell cluster before and after ablation. (C) Heatmap of the differences in pathway activities scored per cell cluster by gene set variation analysis. (D) The expression of HLA-DRB5 in five myeloid cell clusters before and after MWA.

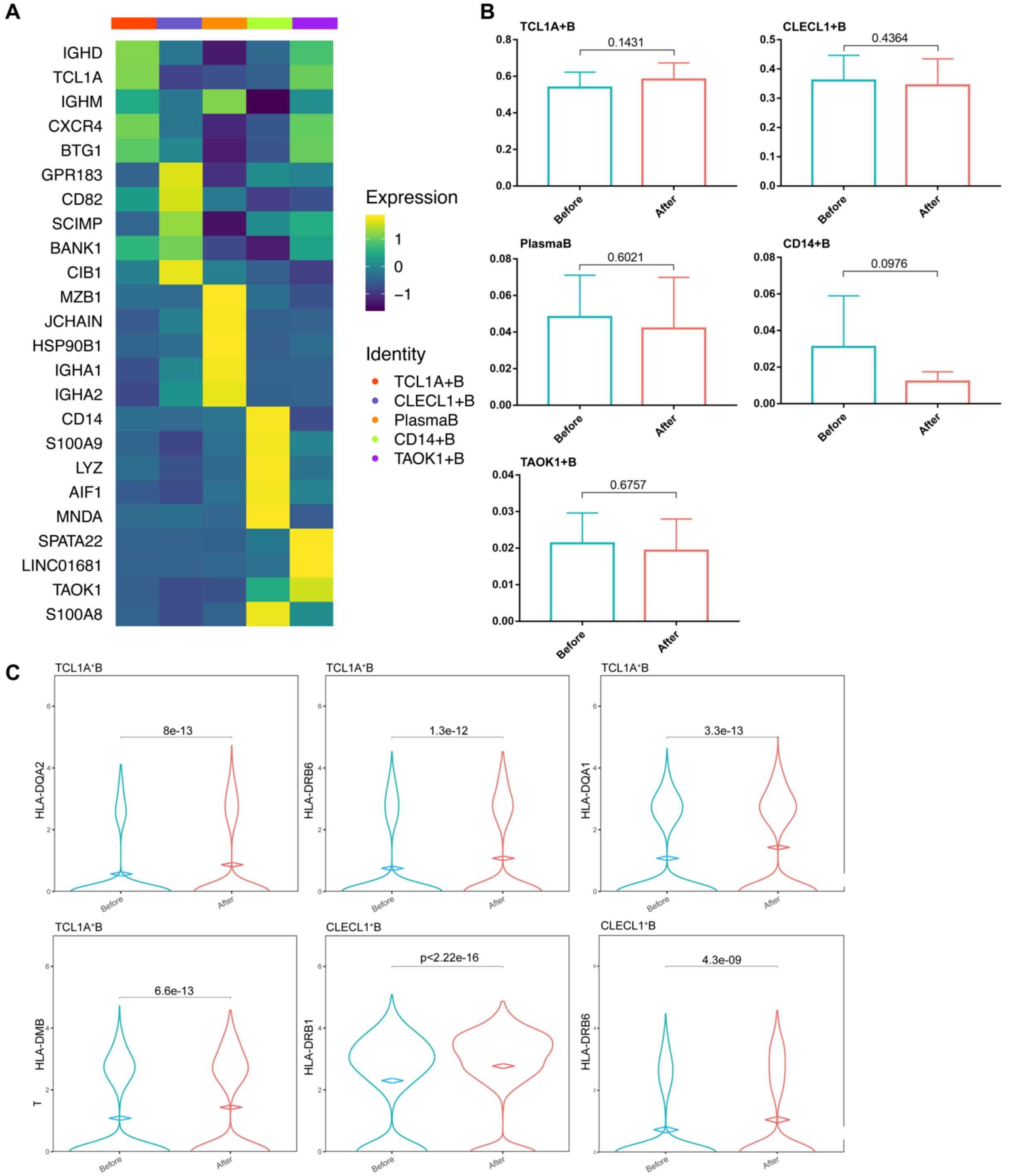

Figure. S5. Changes of the B cells induced by MWA of breast cancer. (A) The heatmap of gene expression in five B cell clusters. (B) The proportions of B cell cluster before and after ablation. (C).The increased expression of major histocompatibility complex molecules in TCL1A+B cells and CLECL1+B cells induced by MWA of breast cancer.

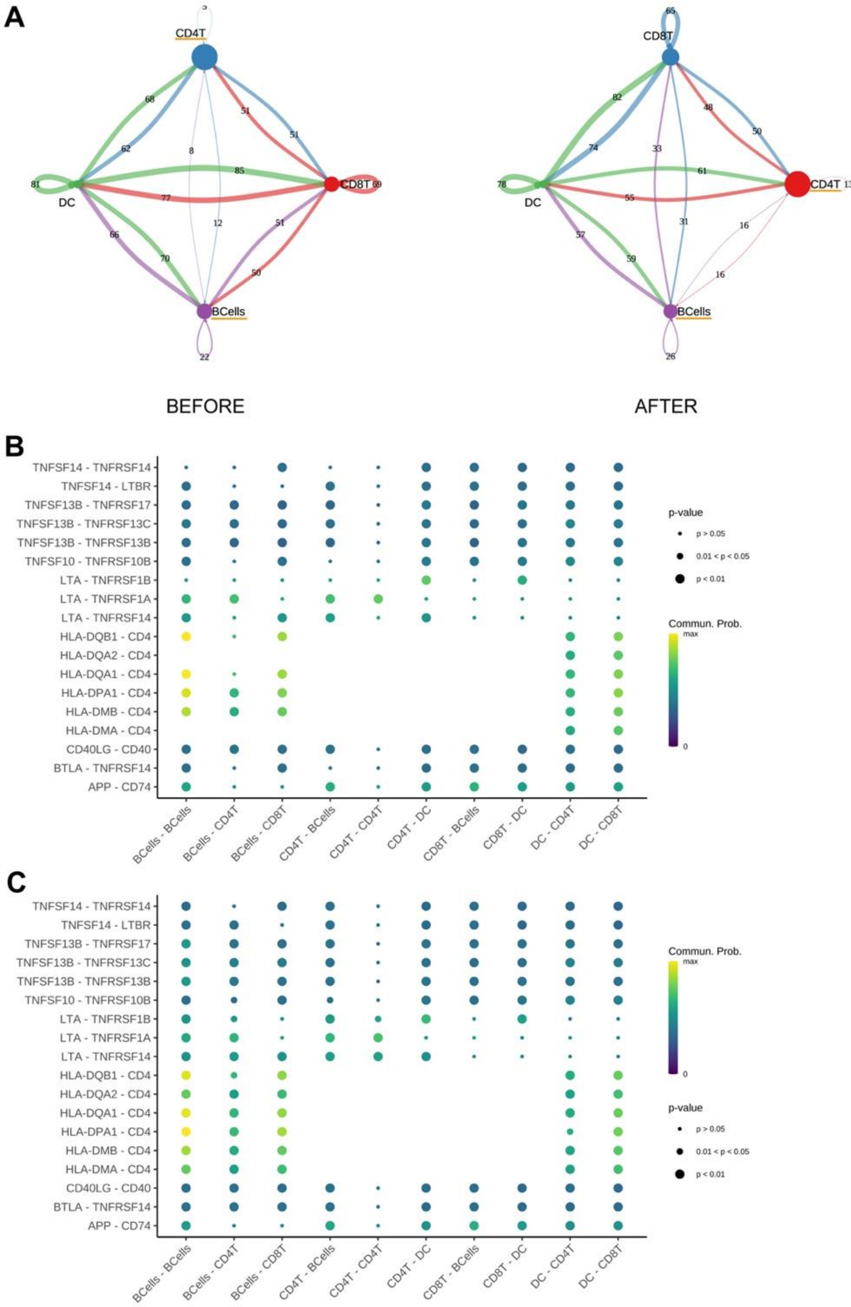

Figure. S6. The cell-to-cell interactions among different cell types by using CellChat before and after microwave ablation in the treatment of breast cancer. (A)The enhanced number of interactions between B cells and CD4+ T cells. (B)The ligand-receptor interactions among different cell types before MWA. (C) The increased ligand-receptor interactions between B cells and CD4+ T cells after MWA. P values indicated by circle size. The means of the average expression level of interactions are indicated by color.

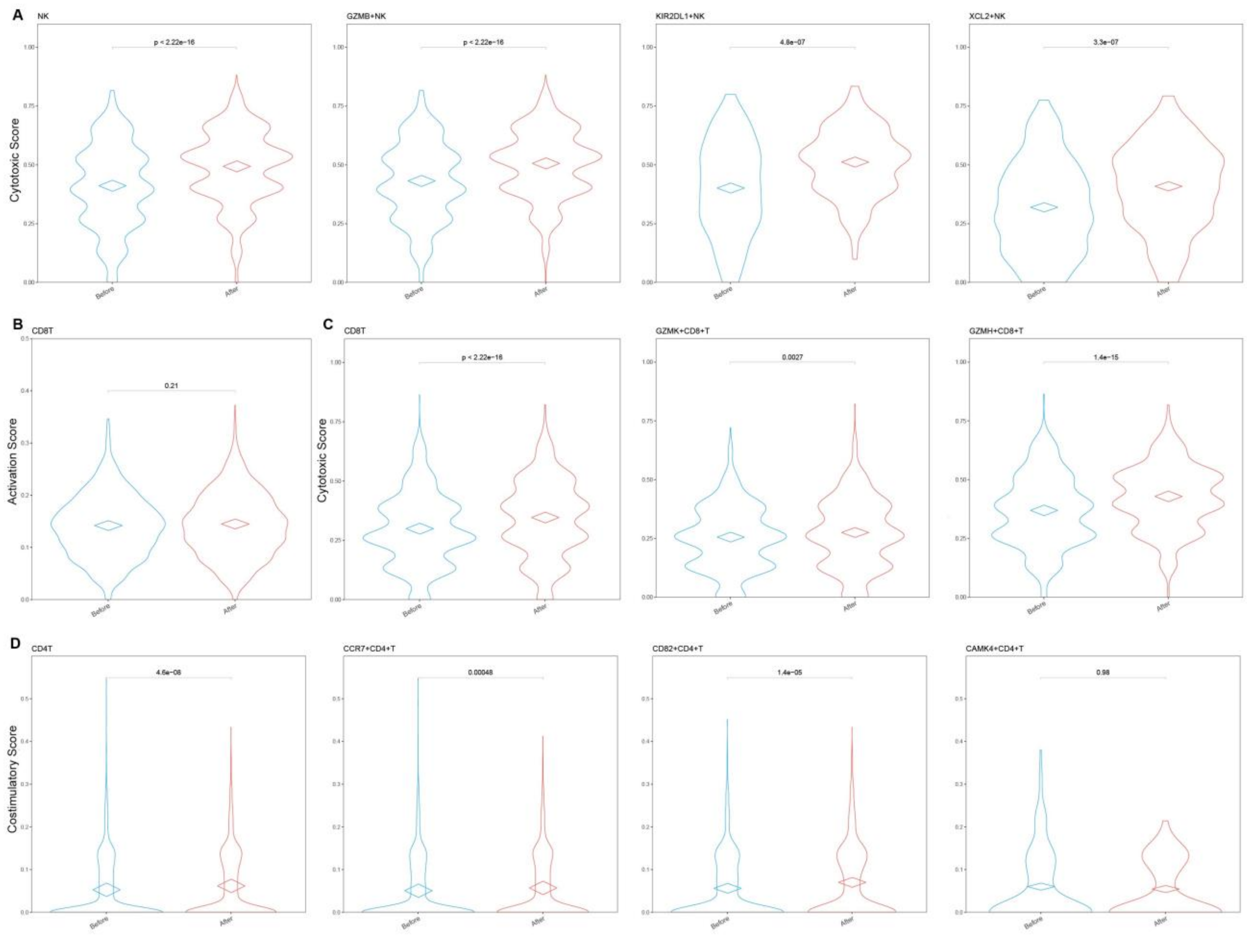

Figure. S7. The biological scores before and after MWA from combination analysis of case 115 and 117 (n=2). (A) The cytotoxic score of NK cells and three subsets of NK cells. (B) The activation score of CD8+ T cells. (C) The cytotoxic score of CD8+ T cells and two subsets of CD8+ T cells. (D) The costimulatory score of CD4+ T cells and three subsets of CD4+ T cells.

Table. S1. The percentages of different immune cells before and after MWA

	**B cells**		**Myeloid cells**		**T & NK cells**	
	Before	After	Before	After	Before	After
All	0.112±0.063	0.104±0.031	0.391±0.217	0.321±0.093	0.496±0.166	0.573±0.105
114 left	0.126±0.060	0.110±0.032	0.331±0.179	0.338±0.094	0.543±0.135	0.551±0.101
115 left	0.111±0.070	0.095±0.021	0.411±0.238	0.301±0.088	0.478±0.179	0.604±0.082
116 left	0.107±0.069	0.106±0.035	0.385±0.243	0.321±0.104	0.507±0.184	0.573±0.118
117 left	0.091±0.038	0.101±0.033	0.452±0.177	0.328±0.103	0.457±0.152	0.571±0.118
118 left	0.122±0.065	0.111±0.030	0.356±0.223	0.299±0.084	0.522±0.172	0.59±0.109
119 left	0.117±0.069	0.105±0.035	0.412±0.237	0.344±0.085	0.471±0.172	0.551±0.1

Table. S2. Baseline characteristics of study population.

**Variables**	**Microwave ablation Total(29)**
**Age, years**	
Range	37-93
Median	69
**Tumor size**	
cT1	15(51.7%)
cT2	14(48.3%)
**ER status**	
Positive	22(75.9%)
Negative	7(24.1%)
Unknown	0(0%)
**PR status**	
Positive	20(69.0%)
Negative	9(31.0%)
Unknown	0(0%)
**HER2 status**	
Positive	4(13.8%)
Negative	25(86.2%)
Unknown	0(0%)
**Molecular subtype**	
Luminal-HER2-negative	20(69.0%)
HER2-positive	4(13.8%)
Triple negative	5(17.2%)
**Local treatment**	
Surgery	0(0%)
MWA	12(41.4%)
MWA followed by surgery	17(55.2%)

cT: clinical tumor stage; ER: estrogen receptor; HER2: human epidermal growth factor receptor 2; PR: progesterone receptor.
